# On the Prevention of Zymotic and Constitutional Diseases: Fevers; Cholera; Consumption, &c.

**Published:** 1865-02

**Authors:** E. D. Mapother

**Affiliations:** Professor of Hygiene, and Medical Officer of Health for the City of Dublin


### Selections.

### ON THE PREVENTION OF ZYMOTIC AND CONSTITUTIONAL DISEASES: FEVERS; CHOLERA; CONSUMPTION, &c.

By Dr. E. D. MAPOTHER, Professor of Hygiene, and Medical Officer of Health for the City of Dublin.

The poison of scarlatina seems the most contagious of all its class, having the greatest infecting distance, and fixing with the greatest tenacity in dwellings, furniture, or clothes—fomites they are technically called. For this reason, when the disease breaks out in schools, it is generally politic to close them up entirely for some time. The heat of boiling water destroys the poison, and rooms or clothes in which the poison may be lurking should be thoroughly steamed or fumigated with sulphurous acid emitted from burning sulphur. The disease is inoculable, but the malady so produced being as severe as the ordinary kind, such an operation is not advisable. The most malignant epidemics of this disease from which Ireland has suffered were in 1801–2–3, and 1834.

Measles poison would seem to be nearly as contagious, for instances have occurred of the disease being carried by children's clothes packed in a box and sent from schools where the epidemic had broken out. In America, this disease has assumed a serious aspect, for 22,000 soldiers were prostrated by it during the first year's campaign. I may mention that Dr. Salisbury, of Ohio, has endeavored to prove that the measles poison owes its origin to a fungus which grows upon rotten straw. He inoculated himself, his wife, and twenty-seven other persons with this fungus, and in all an eruption similar to measles followed, and none of these individuals so protected caught the natural measles, which just at the time was strongly epidemic.

In connection with this subject, I may remark that Dr. H. Kennedy has published an interesting case, where a measles-like eruption followed the application of some mouldy flaxseed meal. Several hundred children, in France, are said to have been inoculated with the disease by inserting a tear from a patient into a puncture.

In my Introductory Lecture, I endeavored to impress on you that typhoid fever was about the most preventible of diseases, yet 140,000 cases occur, and 20,000 at least die of it every year in England, and as the average age of its victims is 21, and all ıanks are obnoxious to it, the very flower of the people is included. I will not dwell on the subject further here, save to remark, that in having pustules on the intestinal mucous surface, it resembles the diseases—small-pox, for example—which are characterized by peculiar eruptions on the skin. It is supposed that the seeds of this disease may dry up, and yet, like those of many plants, may germinate when submitted to favorable conditions. There is much greater risk of contagion from the decomposition of the poison in faulty sewers than from the atmosphere about the patients, and its progress is much more virulent when introduced by water drank than by air inspired. I enumerated many chemical destroyers or disinfectants of acknowledged efficacy, but I wish now to add to the list McDougal's fluid (crude carbolic acid), and McDougal's powder (carbonate of lime), which are cheap, convenient, and thoroughly reliable in eradicating this pest "born of putresence."

Typhus has always been Ireland's epidemic enemy, and is still five times as frequent in Dublin as London, in proportion to the population. You all must have read of the lamentable way in which this fever decimated the army, in past centuries, and the contagion was usually introduced in the following way: Commissions were formerly given to those who collected a certain number of recruits, and a promiscuous rabble, reeking with the seeds of typhus, was often obtained by ransacking the lowest haunts, and even the jails. In these latter places, so virulent was the poison, that the judges, witnesses, and others who were engaged in court, rarely escaped, as I could relate to you, if time permitted, from the writings of Lord Bacon. The intensity of the disease would seem now to be much less, for persons in a house with a typhus patient suffer little risk, and those in neighboring houses none. I have investigated some cases in which the inhabitants of a house were reputed to have caught typhus from patients in a neighboring one; but I have always found that the attack was due to an original focus, usually promoted by similar unsanitary conditions. If there be suitable arrangements for the exit of the infected air and the entrance of the fresh, I have never thought it necessary to banish relatives from the patient's house, for their affectionate solicitude is often more valuable than any hired services. I will now recapitulate the circumstances which recent pathologists assert to be promotive, if not productive, of typhus, and you will acknowledge they are all preventible. 1, overcrowding and defective ventilation; 2, personal squalor, especially the wearing of clothes soaked in cutaneous exhalation, and dark-colored woollen stuffs have by far the greatest power of absording the poison; 3, a low state of system due to scanty or bad food; 4, a medium temperature.

As regards the most plainly malarious of diseases—ague—it used to prevail in Dublin and its vicinity; but owing to improved surface-drainage it has almost wholly disappeared.

The next group of the zymotic diseases is that of which the breathing organs are the seats, and among them influenza is the most clearly epidemic, the most rapidly diffusible, and the most largely fatal, at least 4000 persons in Dublin having succumbed to it in four months of the year 1837. Few old people escape if they are attacked; but Dr. Graves mentions a notable exception. He attended Judge Day, the contemporary of Goldsmith, at the age of 93, and he recovered perfectly. There are reasons for supposing that influenza is due to some specific poison floating in the air, and acting like the emanations from the sweet-smelling vernal grass which gives rise to that remarkable disease, "hay fever."

Whooping-cough, another zymotic, seems to be excited by some poison which acts exclusively on the branches of that widely-distributed nerve, the pneumogastric. That the poison is not solely aërial would appear from a child just born having had the disease, which must have been carried to it in the mother's womb. Quinsy, croup, and diphtheria, are other examples of this group of diseases, and in the prevention of all of them, ventilation, drainage of excreta, and removal of dampness, are hygienic measures of tried and positive efficacy.

Cholera, the most dreaded of the zymotic diseases, which choose as their seat the digestive canal, is, as I endeavored to explain on two previous occasions, communicated by a specific poison emitted from a patient already attacked, and carried to others through the water or air. It, therefore, follows the lines of human intercourse for the most part, but outbreaks do undoubtedly occur which allow of no such explanation. It first appeared in Dublin on 22d March, 1832, and arose in Cork on 12th April, probably from some new source of contagion (perhaps by the steamer which then plied between these cities), for Naas, which lies along the direct road, was not attacked till April 13th. That learned physician, Prof. Aitken, to whose comprehensive treatise I am indebted for many facts, relates the following exceptional case:—"In one of the Western Islands, the most remote from the mainland, the disease suddenly appeared, where so little intercourse existed with the place that the clergyman of the island continued regularly every Sunday, for 18 months, to pray for King William the Fourth, as if he had been alive, after our gracious Queen Victoria had ascended the throne."

The cholera, which is endemic in these countries, prevails, for reasons I explained before, during hot dry weather, and is in London contemporaneous with the putrefaction and stench of the contents of the Thames, and a death in this city, from the same disease, has just been registered by one of our dispensary medical officers. When our water supply will be available, and our sewerage efficient, we may set at defiance even its awfully aggravated and epidemic form.

I will conclude what I have to say upon those zymotic diseases depending upon the introduction of a specific poison into the blood, by mentioning that Prof. Polli, of Milan, has energetically advocated the use of chemial antidotes to them—such as the sulphites—which are known to possess the power of checking catalytic action. If their curative efficacy be proved, they will become still more reliable in preventing the specific zymotic process consequent on the introduction of the poison.

On another occasion, I dwelt at perhaps sufficient length upon the influence of want of pure air in the production of consumption, which is so potent that the deaths among in-door artisans, such as tailors, shoemakers, weavers, and printers, from this disease are at least twice as frequent as among those who labor in the open air. Much may be done in the way of prevention, both by public and private hygienic measures. Under the former head, I include the opening of public parks and grounds, a close supervision of the establishments where people work in numbers, and the application of the most scientific preventives to the special injuries which many noxious trades inflict, and in such arrangements our French neighbors far excel us. Among means which lie in each individual's power are the fit ventilation of bedrooms, to which sunlight should have free access by a proper aspect being chosen, and free exercise in the open air, with the habit of filling the lungs occasionally to the utmost, for by ordinary breathing their upper parts are not inflated, and remember a disused organ always suffers.

I must mention to you some remarkable facts with regard to the habits of those who have afterwards become consumptive—namely, that they have suffered from that kind of dyspepsia in which acid is too freely produced in the stomach, and that they have exhibited a constant dislike for fatty foods. The peculiarity of their disgestion would then be that while albuminous food would be assimilated, the alkalinity of the saliva and pancreatic juice would be neutralized, and their respective functions—namely, the conversion of starch into sugar, and the taking up of fat, interfered with. The remedial powers of cod-liver oil, which insinuates itself so readily into the absorbents, may depend upon their supplying this want. The preventives of consumption are urged so enthusiastically by Dr. McCormac, that I shall sum up by giving you his list—the respiration of a pure untainted atmosphere by day and night—improvement of the artizan's *locale* and habits, including his bent and sedentary posture—the substitution of steam machinery for dry grinding and stone-chiselling—attention to the digestive and cutaneous functions—improvements in the aspects of houses and sleeping-rooms—increase of out-door pursuits—and lastly, full and free respirations in the open air. Dr. Aitken, Professor of Pathology in the Army Medical School, most justly remarks:—

"Experience has now adequately demonstrated, that the tuberculous cachexia springs from causes over which the public, rather than the medical profession, have control. The physician must be at once impressed with the belief, and encouraged with the hope, that when he acquires the confidence of the public in the practice of his profession, he may exercise a powerful influence for good in teaching how much they may themselves control the ravages of consumption by prudent marriages and sanitary attention to offspring. There are several circumstances which show the great influence of public sanitary measures in controlling the ravages of consumption when these measures are scientifically directed to the preservation of the general health, and especially when men are associated together in great communities—an influence much greater than the best directed efforts of the medical profession can establish through their *materia medica.* It is by the mode of life as citizens of the world, in the social relations of husbands and wives, parents and children, and in the public relation of masters and workmen, that the extent and ravages of consumption are to be controlled. It is by a strict attention to the rearing of offspring, and in the subsequent regulation of food, clothing, cleanliness, occupation, the choice of a profession, and by many other circumstances which have an obvious influence (perhaps at first sight inappreciable) on the maintenance of the general health, that our hopes of success as practitioners of medicine must rest in the prevention of that bad habit of body which developes and propagates the tuburcular diseases in civilized society.

Upon dietetic diseases I made a few remarks on a former occasion, and now, concerning one of them—namely, gout—I can tell you in a very few words all the measures which are preventive by quoting Abernethy's reply when asked, What was a cure for the gout? "Live on sixpence a-day and earn it," for temperance, exercise, and a freely acting skin, are among the hygienic requisites comprised in the aphorism.

As I have endeavored to elucidate, our tissues do not remain permanently ours, but are in health being constantly removed and renewed. Now, when renewal fails, and substances lower in the scale of human chemistry become deposited instead, we give the name of "degeneration" to the condition. The fatty form in which a tissue—the muscular, for example—is either chemically converted into fat, or is removed to give place to that substance, is the most frequent, and chooses such vital organs as the heart, brain (yellow softening), and the kidney (chronic Bright's disease). We are warned that sedentary habits are strongly promotive of this affection, for it was fatal to such prolific writers as Abercrombie, Pereira, and Thackery, and such industrious lawyers as Cresswell and Slade. The influence of the ingestion of too much heat-producing food to the exclusion of tissue-material we have already discussed; but it remains for me to explain the physiological effect of alcohol, which we shall find tends powerfully to the production of fatty degeneration. It checks the changes which our tissues are undergoing, interferes with the due oxidation of the blood, or the combustion of its waste material, and, according to many authorities, supplies material itself for the formation of fat, as more certainly does the sugar, which most of its preparations plentifully contain. Distilled spirits are, for this last reason, less promotive of fatty degeneration than other alcoholic liquors. I shall not now dwell on other evils of intoxication, lamentable though they are, save to say that it is not on the individual alone they work destructive changes, for his offspring, in addition to moral injury, undergo most manifest physical deterioration. In this way alcohol is one of the most powerful causes of that remarkable character of disease in the nineteenth century—namely, that while the mean lifetime of adults is becoming prolonged, that of children is being most perceptibly shortened.—*Dublin Medical Press.*

